# Evaluation of Retinal and Optic Nerve Parameters in Recovered COVID-19 Patients: Potential Neurodegenerative Impact on the Ganglion Cell Layer

**DOI:** 10.3390/diagnostics15101195

**Published:** 2025-05-09

**Authors:** Muhammet Kaim, Muhammet Bahattin Kır, Feyzahan Uzun, Hüseyin Findik

**Affiliations:** 1 Department of Ophthalmology, School of Medicine, Recep Tayyip Erdogan University, 53100 Rize, Turkey; feyzahan.ekici@erdogan.edu.tr (F.U.); huseyin.findik@erdogan.edu.tr (H.F.); 2 Department of Ophthalmology, Trabzon Imperial Hospital, 61200 Trabzon, Turkey; bahattinkir@yahoo.com

**Keywords:** COVID-19, optic coherence tomography, optic nerve, retinal nerve fiber layer, retinal ganglion cells

## Abstract

**Background/Objectives:** This study aimed to analyze optic nerve parameters, retinal nerve fiber layer thickness (RNFLT), ganglion cell layer thickness (GCLT), and subfoveal choroidal thickness (ChT) in patients who have recovered from coronavirus disease 2019 (COVID-19). **Methods:** This comparative study included 78 recovered COVID-19 patients (16 men, 62 women) and 56 age- and sex-matched healthy controls (18 men, 38 women). COVID-19 was confirmed in all patients, either through the detection of viral RNA in nasopharyngeal swabs via reverse transcriptase polymerase chain reaction or by serological testing for SARS-CoV-2 antibodies. Spectral-domain optical coherence tomography (SD-OCT) was used to assess optic nerve parameters, RNFLT, GCLT, and ChT. **Results:** The mean age was 35.0 ± 8.3 years in the COVID-19 group and 31.5 ± 8.3 years in the control group, with no statistically significant differences in age or sex distribution between groups (*p* = 0.41 and *p* = 0.16, respectively). Optic nerve parameters and RNFLT (overall and across the four peripapillary quadrants) did not differ significantly between the COVID-19 and control groups. However, the mean ganglion cell–inner plexiform layer (GC-IPL) thickness was significantly reduced in all quadrants in the COVID-19 group compared to the controls. No significant difference was observed in mean subfoveal ChT between groups. **Conclusions:** A significant reduction in ganglion GCLT was observed in recovered COVID-19 patients compared to healthy controls, suggesting a potential neurodegenerative effect of the disease on the optic nerve.

## 1. Introduction

Coronavirus disease 2019 (COVID-19), caused by the severe acute respiratory syndrome coronavirus 2 (SARS-CoV-2), has emerged as one of the most significant global health crises in recent history [1,2]. Initially recognized for its respiratory manifestations, particularly acute respiratory distress syndrome (ARDS), COVID-19 is now understood to be a multisystem disease affecting the cardiovascular, neurological, renal, and ophthalmic systems [3]. The virus has been implicated in widespread endothelial dysfunction, hypercoagulability, and an exaggerated inflammatory response, all of which may contribute to organ damage beyond the lungs [4].

Among its ophthalmic manifestations, ocular surface disorders, including conjunctivitis, are the most frequently reported, with an incidence of approximately 10% [5]. However, increasing evidence suggests that SARS-CoV-2 may also affect deeper ocular structures, including the retina and optic nerve [6,7]. Several case reports and small-scale studies have documented retinal vascular abnormalities, including cotton wool spots, focal hyperreflective dots in the ganglion cell layer, microhemorrhages, and dilated veins, resembling retinal findings observed in systemic vascular diseases such as hypertension and diabetes mellitus [8,9]. Additionally, optic neuritis and other neuro-ophthalmic complications, such as acute macular neuroretinopathy, papillophlebitis and retinitis, have been reported in patients recovering from COVID-19, raising concerns about potential long-term visual impairment [10,11,12,13]. Despite these emerging reports, comprehensive data on the chronic effects of COVID-19 on retinal and optic nerve structures remain limited.

The pathophysiological basis for SARS-CoV-2-related neuro-ophthalmic involvement is not yet fully understood but may be linked to the angiotensin-converting enzyme 2 (ACE2) receptor, which serves as the primary entry point for the virus into host cells. The ACE2 receptor has been identified in various ocular tissues, including the retina, choroid, and retinal pigment epithelium (RPE), suggesting that these structures may be directly susceptible to viral invasion [14]. Beyond direct viral effects, secondary mechanisms such as immune-mediated inflammation, hypercoagulability, and microvascular dysfunction may further contribute to optic nerve and retinal damage [15]. Notably, the pro-inflammatory cytokine storm associated with severe COVID-19 has been implicated in retinal vascular occlusions, raising concerns about possible ischemic complications in the posterior segment of the eye [16].

Optical coherence tomography (OCT) has emerged as a valuable, non-invasive imaging tool for detecting structural alterations in the retina and optic nerve. High-resolution spectral-domain OCT (SD-OCT) allows for precise, reproducible measurements of retinal nerve fiber layer thickness (RNFLT), ganglion cell layer thickness (GCLT), and choroidal thickness (ChT), which are critical parameters in evaluating neurodegenerative and vascular retinal conditions. In systemic diseases, such as multiple sclerosis, diabetes, and hypertension, changes in these retinal layers have been associated with disease progression and prognosis [17], suggesting that OCT findings in post-COVID-19 patients could serve as a potential biomarker for long-term neuro-ophthalmic involvement.

Given the increasing reports of retinal and optic nerve abnormalities in recovered COVID-19 patients, this study aimed to evaluate the structural impact of the disease on the posterior segment of the eye. Using SD-OCT, we assessed optic nerve parameters, RNFLT, GCLT, and ChT in individuals who had recovered from COVID-19 and compared them with age-matched healthy controls. By identifying potential post-COVID-19 retinal and optic nerve alterations, our findings may contribute to understanding the neuro-ophthalmic sequelae of the disease and provide insights into long-term monitoring strategies for affected individuals.

## 2. Materials and Methods

This comparative, cross-sectional study was conducted to evaluate ocular parameters in healthcare workers who had recovered from COVID-19, compared to age- and sex-matched healthy controls. The study adhered to the tenets of the Declaration of Helsinki and received approval from the Kanuni Training and Research Hospital Ethics Committee (Date: 14 March 2021, No.: 23618724). Informed consent was obtained from all participants prior to their inclusion.

This study enrolled 78 healthcare workers (16 men and 62 women) who had recovered from COVID-19 and 56 healthy individuals (18 men and 38 women) as controls. COVID-19 diagnosis was confirmed through reverse transcriptase polymerase chain reaction (RT-PCR) testing of nasopharyngeal swabs or serological detection of SARS-CoV-2 antibodies. Participants in the recovered group had been diagnosed with COVID-19 approximately six months (±2 weeks) prior to the ophthalmic assessments.

The inclusion criteria for both groups were as follows: aged between 18 and 65 years, no history of systemic diseases such as diabetes mellitus or hypertension, and no prior ocular surgeries or trauma. The exclusion criteria encompassed the following: the presence of ocular conditions like glaucoma, cataracts, or retinal diseases, spherical equivalent refractive error exceeding ±3.0 diopters, use of topical ocular medications within the past six months, and pregnancy or lactation.

### 2.1. Ophthalmic Examination

All participants underwent a comprehensive ophthalmologic evaluation conducted by a single experienced ophthalmologist to ensure consistency. The examination included

- Best-Corrected Visual Acuity (BCVA), assessed using a standard Snellen chart.
- Intraocular pressure (IOP), measured with a Goldmann applanation tonometer.
- Anterior Segment Examination, performed using slit-lamp biomicroscopy to evaluate the cornea, anterior chamber, iris, and lens.
- Dilated Fundus Examination, conducted with a 90-diopter lens to assess the vitreous, retina, and optic nerve head.

### 2.2. Optical Coherence Tomography (OCT) Measurements

Spectral-domain OCT imaging was performed using the Cirrus HD-OCT system (Carl Zeiss Meditec, Dublin, CA, USA). Standardized imaging protocols were employed to ensure measurement accuracy and reproducibility. Scans were acquired between 9:00 AM and 12:00 PM to minimize diurnal variations in choroidal thickness. All measurements were conducted by the same experienced examiner to reduce interobserver variability. Only scans with a signal strength of 7 or higher were included; scans with segmentation errors or motion artifacts were excluded. For analysis, the right eye of each participant was selected. If the right eye did not meet the inclusion criteria due to inadequate image quality or segmentation errors, the left eye was used instead.

The following parameters were assessed:

- Peripapillary retinal nerve fiber layer thickness (RNFLT), measured using the Optic Disc Cube 200 × 200 protocol, which provides average thickness and sectoral measurements (temporal, superior, nasal, and inferior quadrants).
- Optic nerve head (ONH) parameters, evaluated using the same protocol, including rim area, disc area, average cup-to-disc ratio, and vertical cup-to-disc ratio.
- Ganglion cell–inner plexiform layer (GC-IPL) thickness, assessed with the Macular Cube 512 × 128 protocol, providing average and sectoral thicknesses.

**Figure 1:**
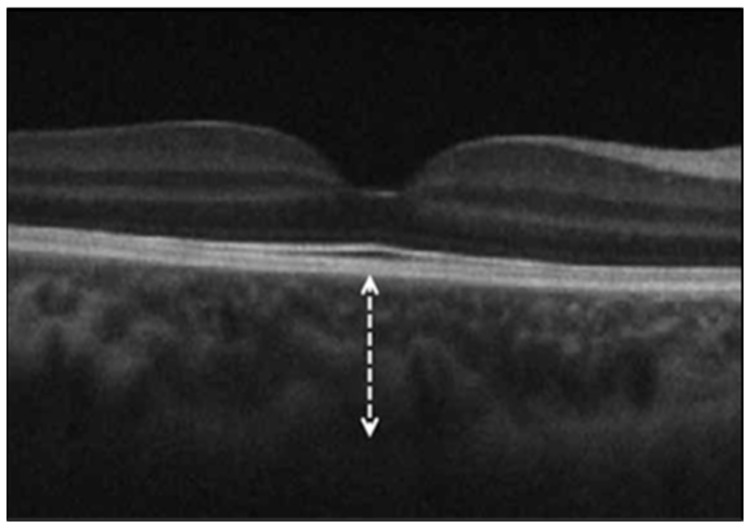
Spectral-domain optical coherence tomography image showing the subfoveal choroidal thickness, measured as the vertical distance (dashed arrow) from the outer border of the retinal pigment epithelium to the inner scleral border (sclerochoroidal interface) beneath the fovea.

Subfoveal choroidal thickness (ChT), measured manually from the outer border of the retinal pigment epithelium to the sclerochoroidal interface using the caliper tool in the OCT software (Zeiss Cirrus Review Software (Carl Zeiss Meditec, version 8.1.0.117)) by two independent masked graders (Figure 1). The average of their values was used for analysis. In cases of disagreement greater than 10%, a third senior grader was consulted.

### 2.3. Statistical Analyses

Statistical analyses were performed using IBM SPSS Statistics for Windows (version 23, IBM Corp., Armonk, NY, USA). Continuous variables are presented as mean ± standard deviation (SD), while categorical variables are expressed as frequencies and percentages. The Kolmogorov–Smirnov test was used to assess the normality of data distribution. Differences in age and sex between the COVID-19 and control groups were analyzed using the independent samples *t*-test and Chi-square (χ^2^) test, respectively. For comparisons of normally distributed continuous variables between the two groups, the independent samples *t*-test was applied. A *p*-value < 0.05 was considered statistically significant.

## 3. Results

This study enrolled 78 healthcare workers who had recovered from COVID-19 and 56 age- and sex-matched healthy control subjects. The mean age was 35.0 ± 8.3 years in the COVID-19 group and 31.5 ± 8.3 years in the control group, with no statistically significant difference (*p* = 0.41). The gender distribution was also comparable between the groups (*p* = 0.16). All participants had a BCVA of 20/20, and IOP measurements were within normal limits.

### 3.1. Optic Nerve Head (ONH) Parameters and Retinal Nerve Fiber Layer Thickness (RNFLT)

Spectral-domain optical coherence tomography (SD-OCT) assessments revealed no significant differences between the COVID-19 and control groups in terms of ONH parameters, including rim area, disc area, average cup-to-disc ratio, and vertical cup-to-disc ratio (*p* > 0.05 for all comparisons) (Table 1).

Similarly, the average peripapillary RNFLT, as well as quadrant-specific measurements (superior, inferior, nasal, and temporal), did not differ significantly between the two groups (*p* > 0.05) (Table 2). These findings suggest that COVID-19 infection does not have a measurable impact on ONH morphology or RNFL thickness in recovered individuals.

### 3.2. Ganglion Cell–Inner Plexiform Layer (GC-IPL) Thickness

In contrast, the mean GC-IPL thickness was significantly reduced in the COVID-19 group compared to the controls across all measured sectors (*p* < 0.05). The average GC-IPL thickness was 78.38 ± 16.21 µm in recovered COVID-19 patients, whereas it was 84.61 ± 8.34 µm in the control group (*p* = 0.012). Additionally, the minimum GC-IPL thickness was notably lower in the COVID-19 group (*p* = 0.014). These results indicate a potential impact of COVID-19 on the inner retinal layers, particularly affecting the ganglion cell complex (Table 3).

### 3.3. Choroidal Thickness (ChT)

The mean subfoveal ChT was 279.4 ± 20.5 µm in the COVID-19 group and 277.3 ± 13.5 µm in the control group. The difference was not statistically significant (*p* = 0.47), indicating that COVID-19 may not have a measurable effect on the choroidal structure in recovered patients.

## 4. Discussion

Our study demonstrated that while optic nerve head (ONH) parameters and retinal nerve fiber layer thickness (RNFLT) remained unaffected in recovered COVID-19 patients, significant reductions were observed in ganglion cell–inner plexiform layer (GC-IPL) thickness across all measured sectors. This suggests a potential impact of COVID-19 on the inner retinal layers. In contrast, subfoveal ChT did not show any significant differences between groups, indicating that choroidal structure may be preserved following COVID-19 infection.

The potential retinal involvement of SARS-CoV-2 can be explained by several mechanisms. One proposed pathway is the infiltration of infected leukocytes across the blood–retina barrier (BRB), facilitating viral entry into retinal tissues [18]. Studies have shown that SARS-CoV-2 can infect and replicate in retinal cells, such as photoreceptors and retinal ganglion cells, further supporting the virus’s potential to directly affect retinal tissues [19]. Another mechanism involves angiotensin-converting enzyme 2 (ACE2), the primary receptor through which the virus induces pathological changes. ACE2 expression is significantly elevated in ocular tissues, including the retinal pigment epithelium (RPE) and choroid [20]. Furthermore, SARS-CoV-2 may directly target the ACE2 receptors of vascular pericytes, contributing to the involvement of the ocular vascular system [21]. Additionally, CD147, a transmembrane glycoprotein known to serve as an alternative receptor for SARS-CoV-2, has been detected at moderate to high levels in retinal and BRB cells [22].

OCT is a non-invasive imaging modality that quantitatively assesses retinal and ganglion cell layers. In the medical literature, it is well-recognized as a valuable tool for diagnosing retinal changes associated with systemic conditions such as diabetes and hypertension, as well as for monitoring disease progression and prognosis. Studies have demonstrated that retinal layer thinning detected via OCT correlates with axonal loss and neurodegeneration in various neurological disorders, where RNFL thinning serves as a marker for neurodegeneration and is associated with increased disability and reduced quality of life [23,24]. Similarly, in post-COVID-19 patients, OCT may serve as a crucial tool for detecting subtle retinal alterations with potential prognostic significance.

Findings regarding RNFLT in post-COVID-19 patients have been inconsistent. The current literature includes numerous studies reporting that the average RNFLT of the optic disc is lower in COVID-19 patients compared to controls [25,26,27]. In a study comparing RNFLT between recovered COVID-19 patients and healthy controls, no significant differences were observed in any quadrant except for sectoral thinning in the nasal quadrant [28]. Conversely, a case series of COVID-19 patients reported increased RNFLT 4 weeks after the diagnosis in seven out of eight eyes, except for one case with pre-existing glaucoma, which showed decreased RNFLT [29]. In another study, post-COVID-19 patients showed statistically significant increases in the global RNFLT [30]. The authors attributed the increased RNFLT to optic nerve inflammation, tissue hypoxia induced by pneumonia, or the effects of COVID-19 treatment. In another study, researchers investigated short-term (1 month) and long-term (12 month) retinal thicknesses, and they observed that the thinning of retinal thickness in the inner and outer rings was temporary, and no difference was obtained at the 12-month follow-up [31]. Ozmen et al. found that the mean and fragmented RNFL and GC-IPL thicknesses measured by OCT were not statistically different in patients who had a moderate disease course and recovered from COVID-19 infection [32]. Similarly, in our study, RNFLT did not differ significantly between the COVID-19 and control groups. We believe that variations in RNFLT measurements may be attributed to the differences in disease severity, treatment protocols, and the timing of OCT evaluations post-infection. The existing literature suggests that inflammation-induced edema and thickening occur in the early phase, followed by thinning and, ultimately, a return to baseline over the long term. Longitudinal studies with extended follow-up will be essential in further elucidating this progression.

COVID-19 can cause microvascular dysfunction and hypercoagulability, leading to reduced blood flow to the retina [33]. This ischemic damage may contribute to GC-IPL thinning, similar to non-arteritic anterior ischemic optic neuropathy or diabetic retinopathy. COVID-19 triggers a hyperinflammatory response (cytokine storm) that can affect the central nervous system, including the retina [34]. The release of pro-inflammatory cytokines (IL-6, TNF-α) may contribute to ganglion cell apoptosis and retinal neurodegeneration. Previous research suggests that GC-IPL thinning may precede axonal loss and that retinal neurodegeneration may occur before vascular alterations [35,36]. Our study demonstrated a significant reduction in GC-IPL thickness across all quadrants in the COVID-19 group. Similarly, Dag Seker et al. reported the thinning of GC-IPL and inner retinal layers in COVID-19 patients, particularly in subjects with headaches [37]. This finding aligns with the neuroinvasive potential of SARS-CoV-2, as previously reported in animal models [38], and neurological complications [39] associated with COVID-19.

Although the reduction in GC-IPL thickness observed in our study was statistically significant, the relative decrease was modest—approximately 7.4%—and falls within the range of normal inter-individual variability reported in healthy populations [40]. While such thinning has been associated with neurodegenerative diseases like glaucoma and Alzheimer’s [41], all participants in our study maintained normal visual acuity and intraocular pressure, suggesting no immediate functional impairment. The clinical relevance of this mild thinning remains uncertain. Additionally, the long-term trajectory of GC-IPL changes following COVID-19 is still under investigation. Some studies suggest that retinal alterations may be transient and resolved within months, while others indicate possible persistence [31,42]. Therefore, further longitudinal studies are necessary to determine whether this thinning represents reversible retinal stress or is an early indicator of lasting neuroretinal damage.

COVID-19 may affect choroidal thickness through multiple mechanisms, primarily driven by the systemic inflammatory response, vascular dysfunction, and microvascular damage associated with the disease. SARS-CoV-2 infection induces a cytokine storm, characterized by elevated levels of pro-inflammatory cytokines such as interleukin-6 (IL-6), tumor necrosis factor-alpha (TNF-α), and interleukin-1β (IL-1β), which can disrupt the blood–retinal barrier and lead to increased vascular permeability in the choroid. Additionally, endothelial dysfunction caused by direct viral invasion via angiotensin-converting enzyme 2 (ACE2) receptors, which are highly expressed in choroidal vasculature, may contribute to altered choroidal perfusion and structural changes [43]. Studies using OCT have reported both increased and decreased ChT in post-COVID-19 patients. Increased ChT may result from inflammatorily induced choroidal vascular dilation and leakage, whereas thinning could reflect ischemic damage and hypoperfusion secondary to endothelial dysfunction and thrombotic microangiopathy. Some research indicates an increase in ChT among individuals recovering from COVID-19. For instance, in one study, central foveal choroidal thicknesses were found to be higher in COVID-19 patients compared to healthy controls [44]. In another study, ChT was found to be increased during the active phase of COVID-19; however, no significant difference was observed between recovered patients and the control group during the post-recovery period [45]. Conversely, Erdem et al. [46] observed a decrease in the thickness of the choroidal tissue in all measured areas in recovered COVID-19 patients. In another study, a decrease in CT during the early post-infectious period was reported, and this choroidopathy was recovered from during the follow-up period [47]. In our study, we were not able to find a measurable effect of COVID-19 on the choroidal structure in recovered patients. Although our study did not demonstrate a significant difference in subfoveal ChT, the mixed findings in the literature suggest that choroidal alterations may occur in some individuals, possibly reflecting systemic vascular and inflammatory responses to COVID-19. We believe that these discrepancies may stem from differences in study design, patient populations, timing of measurements post-infection, individual vascular reactivity, and the severity of the disease.

The main limitations of this study include a relatively small sample size, its single-center cross-sectional design, which precludes longitudinal assessment of retinal changes, and the absence of a long-term follow-up. Additionally, baseline ophthalmological evaluations were unavailable, making it difficult to determine whether the observed retinal alterations predated the infection.

Understanding the ocular effects of COVID-19 is crucial, as retinal alterations may indicate broader neurovascular dysfunction. Our findings highlight the need for comprehensive ophthalmic evaluations in recovered patients and suggest that follow-up OCT assessments should be considered for persistent visual disturbances due to the risk of long-term neuro-ophthalmic sequelae. Future research should prioritize large-scale longitudinal studies to track retinal changes over time and explore therapeutic strategies, such as neuroprotective or anti-inflammatory treatments, to mitigate retinal damage. Additionally, investigating correlations between retinal findings and systemic inflammatory markers could provide further insight into the underlying pathophysiology.

In conclusion, this study compared RNFL and ganglion cell layer thickness between recovered COVID-19 patients and healthy controls. While no significant differences were found in RNFLT, a significant reduction in GCL-IPL thickness was observed, suggesting the potential early retinal neurodegenerative effects of COVID-19. Further longitudinal studies with larger cohorts are warranted to clarify the long-term impact of SARS-CoV-2 on retinal health. These findings highlight the need for continued ophthalmic monitoring in post-COVID-19 patients, as well as further research to determine the long-term impact of SARS-CoV-2 on retinal and neuro-ophthalmic health.

## Figures and Tables

**Table 1:** Comparison of ONH parameters between COVID-19 and healthy control subjects.

	Control Group	COVID-19 Group	*p* Value
ONH parameters			
Rim area (mm^2^)	1.48 ± 0.29	1.45 ± 0.26	0.433
Disc area (mm^2^)	1.89 ± 0.27	1.87 ± 0.32	0.606
Average C/D ratio	0.42 ± 0.15	1.41 ± 0.18	0.741
Vertical C/D ratio	0.40 ± 0.14	1.38 ± 0.18	0.596

ONH = optic nerve head; C/D = cup-to-disc ratio; *p* < 0.05 indicates statistical significance (independent two-sample *t*-test).

**Table 2:** Comparison of RNFL thickness between COVID-19 and healthy control subjects.

	**Control Group**	**COVID-19 Group**	***p* Value**
RNFL Thickness (μm)			
Average	96.42 ± 10.19	93.16 ± 9.89	0.071
Superior	119.66 ± 13.30	122.69 ± 17.96	0.267
Inferior	122.28 ± 17.11	127.10 ± 15.44	0.093
Nasal	68.07 ± 9.38	70.75 ± 13.03	0.172
Temporal	67.08 ± 15.88	65.90 ± 11.57	0.622

RNFL = retinal nerve fiber layer; *p* < 0.05 indicates statistically significance (independent two-sample *t*-test).

**Table 3:** Comparison of GC-IPL thickness between COVID-19 and control subjects.

	Control Group	COVID-19 Group	*p* Value
GC-IPL Thickness (μm)			
Average	78.38 ± 16.21	84.61 ± 8.34	0.012
Minimum	74.36 ± 18.41	81.42 ± 10.31	0.014
Superior	78.50 ± 17.68	85.82 ± 8.84	0.007
Superonasal	79.50 ± 16.00	86.12 ± 10.13	0.010
Superotemporal	76.83 ± 16.91	84.03 ± 8.24	0.005
Inferior	77.00 ± 16.13	82.71 ± 8.89	0.022
Inferonasal	79.49 ± 15.54	84.73 ± 8.93	0.030
Inferotemporal	78.74 ± 16.77	84.53 ± 8.37	0.023

GC-IPL = ganglion cell–inner plexiform layer; *p* < 0.05 indicates statistical significance (independent two-sample *t*-test).

## Data Availability

The original contributions presented in the study are included in the article, and further inquiries can be directed to the corresponding authors.
